# Bacteriophages and the One Health Approach to Combat Multidrug Resistance: Is This the Way?

**DOI:** 10.3390/antibiotics9070414

**Published:** 2020-07-16

**Authors:** Mary Garvey

**Affiliations:** Department of Life Science, Sligo Institute of Technology, Sligo, Ireland; garvey.mary@itsligo.ie; Tel.: +353-071-9305-529

**Keywords:** multi drug resistance, infectious disease, bacteriophages, One Health, re-emerging

## Abstract

Antimicrobial resistance necessitates action to reduce and eliminate infectious disease, ensure animal and human health, and combat emerging diseases. Species such as *Acinetobacter baumanniii*, vancomycin resistant *Enterococcus*, methicillin resistance *Staphylococcus aureus*, and *Pseudomonas aeruginosa*, as well as other WHO priority pathogens, are becoming extremely difficult to treat. In 2017, the EU adopted the “One Health” approach to combat antibiotic resistance in animal and human medicine and to prevent the transmission of zoonotic disease. As the current therapeutic agents become increasingly inadequate, there is a dire need to establish novel methods of treatment under this One Health Framework. Bacteriophages (phages), viruses infecting bacterial species, demonstrate clear antimicrobial activity against an array of resistant species, with high levels of specificity and potency. Bacteriophages play key roles in bacterial evolution and are essential components of all ecosystems, including the human microbiome. Factors such are their specificity, potency, biocompatibility, and bactericidal activity make them desirable options as therapeutics. Issues remain, however, relating to their large-scale production, formulation, stability, and bacterial resistance, limiting their implementation globally. Phages used in therapy must be virulent, purified, and well characterized before administration. Clinical studies are warranted to assess the in vivo pharmacokinetics and pharmacodynamic characteristics of phages to fully establish their therapeutic potential.

## 1. Introduction

Antimicrobial resistance (AMR) and multidrug resistance (MDR) are increasing issues globally, as more microbial species (bacteria, virus, fungi, protozoan) gain resistance to currently available therapeutic options. As such, issues increasingly arise relating to prolonged infectious disease, increased mortality, biosecurity issues, animal health, and foodborne disease outbreaks. Indeed, the issue is so great that different classifications have been established for MDR species, where extensively drug-resistant (XDR) species are only susceptible to 2 antimicrobial drug categories and pan-drug-resistant (PDR) species are resistant to all antimicrobial drug categories [1] currently available. Additional negative impacts relate to the economic burden domestically and nationally inflicted by AMR. According to the World Health Organization (WHO), MDR infectious disease will result in 10 million deaths annually by 2050, with approximately 24 million people forced into poverty by 2030 [2]. At present, 0.7 million people die annually from drug-resistant disease, with 230,000 deaths directly related to MDR tuberculosis (TB) alone. Indeed, the WHO reports that of the TB cases in 2014, an estimated 3.3 percent were multidrug resistant [3]. The public health cost of AMR, and in particular antibiotic resistance (ABR), is expected to be significant, with over 2 million people affected annually [4]. It is estimated that ABR infections and complications cost €9 billion annually in Europe [5] alone. Bacterial species such as carbapenem-resistant Gram-negative *Klebsiella pneumoniae*, *Escherichia. coli, Acinetobacter baumannii*, and *Pseudomonas aeruginosa*; and Gram-positive methicillin-resistant *Staphylococcus aureus* (MRSA), vancomycin-resistant Enterococcus (VRE), and *Streptococcus pneumoniae* are frequently associated with high morbidity rates due to limited treatment options [6]. Fungal pathogens are also a growing problem for public health in terms of untreatable infectious disease, as fungal infections have become more prevalent in immunodeficient persons [7]. Fungal species often possess intrinsic and acquired resistance to numerous antifungal drugs, making them MDR. The fungal species commonly associated with invasive mycosis are *Candida albicans, Candida krusei*, *Candida auris*, *Aspergillus fumigatus*, and *Cryptococcus neoformans* [8]. *Candida auris* is an emerging MDR nosocomial pathogen associated with high mortality [9]. At present, there is debate relating to the potential of *Archaea* to stimulate gastrointestinal and metabolic diseases, such as colorectal cancer, inflammatory bowel disease, irritable bowel syndrome, constipation, and obesity in humans [10]. However, no definitive causation has been identified. As a human and animal intestinal colonizer, its role in morbidity requires further investigation.

At present, there is a global drive to identify, develop, and manufacture novel antimicrobial agents for use in fighting this AMR pandemic [11]. Novel areas being researched include the use of bacteriocins (ribosomally synthesized antibacterial peptides) [12] and bacteriophages (viruses infecting bacterial species), amongst other approaches. Phage therapy using bactericidal viruses termed bacteriophages shows potential in the treatment of AMR infectious disease. These viruses are strict bacterial parasites that bind to bacterial surface receptors and inject their nucleic acid [13], hijacking the bacterial cell to reproduce, subsequently causing cell lysis and death. Importantly, legislation aimed at reducing and limiting the use of antimicrobial drugs has been developed and implemented. The “One Health” approach is one such initiative operated under the guidance of the One Health Commission, a globally focused organization. The aim of One Health is to protect human health by protecting animal and environmental health, biodiversity, food safety, and food security, amongst other interrelated areas. This review aims to outline the potential for bacteriophages to act as antimicrobial agents for use in line with One Health. This descriptive review outlining bacteriophages and their application, limitations, and development will inform readers on a promising area of research and pharmaceutical development.

## 2. One Health

The significant increase in human populations, domestic animal populations, and food-producing animal populations; the global decline in wild animal populations; and widespread environmental damage has promoted the occurrence of emerging and re-emerging diseases. Environmental changes, agricultural intensification, climate change, and the destruction of wildlife habitats are also factors promoting the emergence of zoonotic diseases [14], which transmit from animals to humans. Furthermore, environmental pollution with toxic chemicals, including pesticides, fertilizers, industrial chemicals, and antibiotic-resistant genes, threatens both human and animal populations [15]. The One Health approach initially emerged internationally with the aim of addressing emerging, re-emerging, and zoonotic diseases [16]. As the concept grew, however, it was expanded to include environmental health (ecosystems) and sustainability, with a clear understanding of the relationship with human health. Currently, One Health is a transdisciplinary approach implemented in many interdependent areas, integrating human health, animal health, plant health, environmental health, and biodiversity. Preventative medicine, active surveillance, knowledge transfer, and multiple transdisciplinary collaborative efforts are key aspects of a successful One Health approach. Knowledge transfer is a dynamic exchange of knowledge between the medical practitioners and researcher producers and may be achieved via dissemination of research findings, conference proceedings, and recognized frameworks established by governing bodies such as the Center for Disease Control (CDC) or Food and Drug Administration (FDA). Undoubtedly, at present AMR is one of the biggest threats to animal and human health, where emerging microbial species possess intrinsic and acquired resistance to antimicrobial therapy.

### One Health, Zoonosis, and MDR

Antimicrobial therapeutics have been one of the most beneficial medical discoveries to date. The synthetic and semi-synthetic production of these natural antibacterial compounds has provided an effective means of controlling infectious disease for decades. Unfortunately, antibiotic resistance is also a natural survival mechanism for assaulted species, which has been proliferated by anthropogenic use in human and veterinary applications. Antibiotic use is predominant in two areas—in human medicine and in food-producing animals for growth promotion and prophylaxis [17]. Resistance to antibiotics occurs via alteration of antibiotic targets, mutation in target genes and efflux pumps, and enzymatic degradation, and can be intrinsic or acquired via plasmids and horizontal gene transfer (HGT). The excessive proliferation of resistance in emerging and re-emerging species relates to selective pressure from misuse, overuse, and environmental pollution with constant low levels of antibacterial compounds. Indeed, studies report that up to 50% and 80% of antibiotic use in humans and animals, respectively, is not necessary [17]. Consequently, antibiotic resistance genes (ARGs), which are a natural element in all environments, have now become an environmental contaminant due to excessive anthropogenic activity. The environmental presence of these genes further proliferates AMR species in soil and water, as genes are incorporated and shared via plasmids, transposons, and by horizontal gene transfer (HGT) [11]. AMR infections are associated with increased morbidity rates, prolonged morbidity, metastatic bacterial infections, disease recurrence, and future opportunistic infections. This is increasingly evident with the prevalence of nosocomial infections (Table 1) resultant from MDR and XDR species of *E. coli, K. pneumoniae,* MRSA, *C. difficile*, VRE, *P. aeruginosa*, and *Salmonella* sp., amongst others. These species are frequently associated with therapeutic failures, increased risk of complications, deteriorating pathological conditions, and mortality [18]. Furthermore, there is a lack of development of novel antibacterial drugs for treating MDR Gram-negative species, mainly those producing carbapenemases, where no current antibiotics are effective [5]. One health aims to address the issue of AMR mainly by reducing the use of antibiotics in food-producing animals [19]. However, natural waterways and wildlife species are also important reservoirs for resistant organisms and ARGs, as livestock fecal waste, drug disposal, and aquaculture are routes of contamination. Zoonotic disease accounts for over 60% of infectious disease in humans. “Animal to human” (and “human to animal”) transmission occurs from co-habiting, food production, and contact with animal feces. This interconnection of humans and animals via shared ecosystems and the food chain is now an area that is considered under the One Health approach [3]. Controlling animal disease and zoonotic pathogens is effective in controlling human disease, as seen with human brucellosis, which is reduced when controlled in the animal reservoir. Brucellosis is a highly contagious bacterial disease of cattle and a foodborne zoonotic pathogen associated with chronic symptoms in humans.

Zoonotic diseases negatively impact society by reducing the quantity and value of the produce from livestock, decreasing trade and productivity, and increasing financial expenditure on disease control and treatment and environmental pollution with AMR and ARGs. In food production, for example, excessive use of antibiotic therapy has been correlated with AMR *E. coli* isolated from poultry, dairy, and pig farming, with resistance genes to tetracycline, quinolones, aminoglycosides, vancomycin, and β-lactams also detected in animal species [49]. Resistance in *Salmonella* and *Campylobacter* is considered is a major concern in food-producing animals, where resistance to fluoroquinolones or third-generation cephalosporins represents a major risk [50], as both antibiotics are limited therapeutic options for serious *Salmonella* and *E. coli* infections in humans [2]. Sulfonamides, for example, were once the first-line drugs used to treat salmonellosis and UTIs in nosocomial situations. The WHO has classified certain antibacterial drug classes as critically important antimicrobials (CIA) for human medicine, including quinolones, sulfonamides, tetracyclines, cephalosporins, polymyxin, and aminoglycosides [51]. The World Organization for Animal Health (OIE) also created a CIA list of antimicrobials that are important in veterinary medicine, termed veterinary critically important antimicrobial agents (VCIA). While the aim was to use different antibiotic classes for human and animal therapies to reduce AMR as part of One Health, there is some overlap between both systems, such as the macrolides, aminoglycosides, cephalosporins (third-generation), and polymyxins, which are listed as both WHO CIA and VCIA. The ESKAPE pathogens (*Enterococcus faecium, Staphylococcus aureus, Klebsiella pneumoniae, Acinetobacter baumannii, Pseudomonas aeruginosa,* and *Enterobacter species*) are the leading cause of nosocomial infections globally [52]. The isolation of MRSA, VRE, and extended-spectrum β-lactamase (ESBL)-producing *E. coli, K. pneumoniae,* and *A. baumannii* strains from humans, livestock, and contaminated food indicate livestock as a source of these clinically relevant bacteria. *A. baumannii* strains have been isolated from animals, including poultry, livestock, horses, and domestic animals, and are mainly associated with outbreaks in veterinary clinics or hospitals [49]. The majority of ESKAPE isolates are MDR, as they share genetic material via HGT, and as such are associated with the highest risk of mortality [53] of the human bacterial infectious diseases.

The emergence of AMR ESKAPE bacteria is associated with the inappropriate use of antibiotics, followed by distribution into the environment and transfer to animals and humans [54]. ESKAPE pathogens include enteric organisms and soil commensals (*Pseudomonas* and *Acinetobacter*), which are prevalent in food-producing animals, slaughterhouse wastewater, and surrounding environments. The emergence of ESBL-producing or fluoroquinolone-resistant Enterobacteriaceae in healthcare settings and in the poultry production chain is one example of MDR pathogens of zoonotic nature [54]. The use of virginiamycin as a growth-promoting antibiotic in poultry has been linked to the development of resistance to streptogramin combinations in *Enterococcus* species [49]. An externality of AMR infectious disease relates to iatrogenic disease states in animals and humans, where the treatment of resistant bacterial infection results in co-morbidities in the host. Morbidities associated with antibiotic-induced (pseudomembranous) colitis, inflammatory bowel disease (IBD), dysbiosis, leaky gut syndrome, irritable bowel syndrome (IBS), and autoimmunity are associated with prolonged antibiotic therapy, as seen in AMR cases [55]. Alterations in the gut microbiota termed dysbiosis have also been associated with the pathogenesis of systemic conditions, including obesity, metabolic syndrome [56], and systemic rheumatic diseases such as rheumatoid arthritis, systemic lupus erythematosus, systemic sclerosis, ankylosing spondylitis, and Sjögren’s syndrome [57].

## 3. Bacteriophages

One health calls for interdisciplinary research into combating the issue of AMR and encourages the development of novel treatment options to reduce or replace antibiotic agents in human and animal medicine. Certainly, one area showing potential is the use of phages to combat bacterial infectious disease. Such phages are viral species that specifically infect bacteria by injecting their genetic material into the host bacterial cell. They are ubiquitous in aquatic and terrestrial ecosystems and in the microbiome of animal species. In humans, phages are present on body surfaces, such as skin, oral cavity, lungs, intestines, and the urinary tract, where they act as a natural predator to the bacterial microbiome [58]. Commensal phages outnumber bacteria, where they aid in controlling and regulating bacterial communities, preventing dysbiosis. Approximately 96% of these viruses are tailed phages [59] within nine families in the order Caudovirales, including *Myoviridae, Siphoviridae*, and *Podoviridae* [60,61]. The remaining 4% are non-tailed phages, of which 190 have been identified to date. Phages lacking a tail are morphologically grouped into 3 types, consisting of 10 families—those having polyhedral capsids, filamentous phages showing spiral symmetry, and phages with diverse capsids [62]. As with all viruses, they are unable to replicate outside of a host cell and are intracellular obligate parasites. Phages consist of either single- or double-stranded DNA or RNA (Table 2) protected by a protein capsid [63].

Phages bind to a range of host surface receptors, such as carbohydrates, lipopolysaccharides, teichoic acid, pili, and proteins [58], using bacteriophage antireceptors located at the tip of the viral tail. This receptor interaction promotes viral specificity for certain bacterial species. Studies suggest that there are approximately 10 different phages for each bacterial cell (virus bacteria ratio of 10 to 1), with some recognizing only one receptor type (monophages) and others recognizing a range of receptors (polyphages) on the cell [65], demonstrating further specificity. As such, one phage may be specific for one host bacterial species or strain. Indeed, this viral specificity allows phages to be routinely used in epidemiological studies to identify pathogenic agents of infectious disease isolated from clinical samples [63]. These viruses must penetrate the host cell envelope, and therefore are equipped with enzyme endolysins (lysins) and virion-associated peptidoglycan hydrolases capable of piercing the capsule and peptidoglycan layer of their specific bacterial host [68]. Once the genetic material has been injected into the bacterial host, one of the following scenarios may occur: (1) bacterial cell lysis and release of new phages (lytic cycle); (2) release of new phages by extrusion without cell lysis over generations (filamentous); or (3) the phage may reside in the bacterial cell as a plasmid or integrated into the chromosome (temperate or lysogenic) [63]. Lytic phages (virulent phages) reproduce within the bacterial cell and emerge using lytic enzymes (holins), causing cell lysis and destruction, releasing viral progenies. RNA phages, including the *E. coli* phages MS2 and Qβ (Table 2), are examples of such virulent phages [69].

Temperate phages induce either a lytic or a lysogenic lifecycle, where the phage integrates its genetic material into the host genome or forms circular or linear plasmids within the host cytoplasm [70]. This lysogen (bacterial cell with concomitant viral genes) establishes a stable relationship with the host, allowing the virus to reproduce indefinitely during bacterial cell division [69]. The insertion of viral genes into bacterial species promotes genetic variability, metabolic functions, provides protection against other phages, and provides the bacteria with mobile genetic elements, including resistance genes. Phages have an important function in the evolution and pathogenesis of bacterial species, and as such may provide a means of combating AMR. Phages are also essential to the environmental ecology, impacting the cycling of organic matter in the biosphere [71], bacterial diversity, and biogeochemical cycles. Given their ecological importance and functions as genetic resources within the environment [72], phages may represent an untapped source of novel medical therapeutic options. Viral metagenomics based on genetic sequencing technology is a valuable tool in determining phage diversity [73] and viral load in environmental, animal, and human ecosystems, and allows researchers to identify new phage therapy options. Phages of bacterial species associated with human disease are being isolated from the human environment itself, wastewater, and environmental sources. For example, Xu et al. investigated a phage isolated from wastewater that demonstrated lytic activity against multidrug-resistant *E. coli* strains [74].

### 3.1. Phages as Human Therapy against Zoonotic Pathogens

Due to their vast environmental and animal abundance, phages have been associated with the contamination of food such as dairy (contributing to AMR zoonosis) [71], and even commercially available vaccines and sera [75]. Phage therapy for controlling infectious disease can be bactericidal (lytic phages) or bacteriostatic and can be applied as local or systemic therapy [76]. Studies have reported on the efficacy of phages against ABR ESKAPE pathogens and *Streptococci, Proteus, Salmonella*, *Shigella, Serratia, Campylobacter, Listeria*, and *Yersinia* [62,64,77]. The investigated disease states included suppurative wound infections, gastroenteritis, sepsis, osteomyelitis, dermatitis, empyemas, and pneumonia [62]. Phage treatment of ABR *Pseudomonas aeruginosa* infections in cystic fibrosis patients is one area where phage therapy may provide significant patient care [78]. The investigations of Jennes et al. (2017) demonstrated the efficacy of BFC1 phage therapy against *P. aeruginosa* septicemia in a 61-year-old man, where negative blood cultures were obtained following therapy, with no adverse side effects being evident [79]. Similarly, a study by Duplessis et al. reported on the phage treatment of a 2-year-old boy with a history of DiGeorge syndrome, complex congenital heart disease, and *P. aeruginosa* bacteremia. A phage cocktail was administered to the patient, eliminating the bacteremia and subsequently producing negative *Pseudomonas* blood cultures [80].

The Eliava Institute in Georgia has reported a significant improvement in patients treated with staphylococcal phages. Indeed, staphylococcal phages have been used to successfully treat infections where antibiotics have failed; for example, diabetic patients suffering with MRSA-infected foot ulcerations were healed with phage therapy when amputation was the only alternative option [81]. The modification of phage genomes may also guide the research and development of safer lytic phage therapy and the expansion of their host range [58]. The use of bacteriophage-encoded peptidoglycan hydrolases and lysins for topical and systemic therapy is also an area of ongoing research [68]. Indeed, phage lysis proteins (enzybiotics) may offer some advantage over phages, as they have a broader host range, are effective on multiple species, do not transmit virulence genes (e.g., ARG), and the bacteria possess limited resistance [68]. Microbial biofilms on indwelling medical devices and tissues represent a major issue, as biofilm communities are AMR and difficult to eradicate. Studies report that phages also appear to prevent biofilm formation and to kill biofilm communities, as they produce enzymes that degrade the extracellular polymeric substance (EPS) of the biofilm matrix [62]. Phages can permeate the EPS matrix that binds macromolecules and cells to eliminate their target bacterial cells [82], leading to biofilm degradation. Studies demonstrated the activity of phage T7 modified to express the biofilm-degrading enzyme, dispersin B, which effectively reduced viable *E. coli* cells more so than the unmodified T7 phages [58]. Additionally, phage genetic modifications generating phage artilysins (synethic endolysins), which degrade LPS peptides, show potential for activity against *Salmonella ser. Typhimurium*, *Pseudomonas aeruginosa*, and *Acinetobacter baumannii* [83]. Such studies illustrate the potential of phages and phage enzymes for the treatment of zoonotic-resistant pathogens.

Bacteriophages offer numerous advantages over conventional antibiotics for treating infectious disease. Specifically, these advantages include bacterial specificity, potency, limited immune response in the host, self-propagation while also being self-limiting, single administration, and being amenable to bioengineering, giving them undeniable potential as the way forward in combatting AMR. Phage specificity for host bacterial species is a major advantage [65] once the causative agent of infection has been identified or when phage cocktails are implemented. This specificity also means that bacteriophage treatment does not upset the natural microbiome of the patient, as seen with antibiotics, which induce dysbiosis. For lytic phages, repeated treatment is not needed as the virus self-propagates and continuously infects the bacterial cells until complete bacterial cell death has occurred. Therapeutic success is, therefore, achieved by complete elimination of the etiological agent of disease. Once pathogen cell numbers have been eliminated, the phages are self-limiting and should not cause any immune reaction in the host. Indeed, the presence of a phage titer may induce an immune response [84] within the patient to the infectious bacterial species, encouraging a more rapid elimination of infection. Immune stimulation occurs when phages bind to universal molecular patterns associated with pathogen-associated molecular patterns (PAMP) [85], leading to bacterial elimination in the patient. Clinical trials assessing this phage bacterial interaction are varied, however, where variation in the studies demonstrate either complete bacterial clearance or no bacterial clearance in the patient. [86]. Disadvantages associated with phage therapy include phage specificity, which may require bacterial pathogens to be identified prior to phage administration; phage efficacy, which may be limited to only one subtype of bacterial pathogen [62]; and phage administration, which is associated some challenges that must be overcome. Bacterial resistance to phages is also an issue and may be overcome by use of multiple phage or phage–antibiotic combinations. Bacteria gain phage resistance by alteration of phage-binding receptors or the development of adaptive immunity [87].

Successful phage therapy is dependent on the pharmacodynamic and pharmacokinetic properties of the phage in vivo [88]. Phage kinetics relates to numerous critical parameters, including the phage adsorption rate, latency period, initial dosage, phage elimination, phage resistance, and phage distribution in the human body [65]. Additionally, bacteriophage-mediated cell lysis may promote lipopolysaccharides (LPS) endotoxin release from Gram-negative bacterial species, which can stimulate immune reactions in the patient and lead to an inflammatory response [89] or sepsis. Such LPS release and inflammatory reaction in the patient, however, can also occur with the prolonged use of bactericidal antibiotics. Phage formulation is an important consideration, as the route of delivery offers obstacles to overcome in delivering phage therapy. Oral delivery, for example, leads to acidic exposure in the stomach with the presence of food-bolus-absorbing virions. Local and topical treatments require the formulation of gels and creams containing emollients, which may trap viral particles and prevent contact with bacterial species. Formulation stability also relates to light exposure, temperature, preservatives, the ionic nature, pH, and the overall electrostatic charge of the phages [58]. Temperate phages as antibiotic therapeutics are problematic, as these phages often display superinfection immunity, where bacteria become immune to the virus. Additionally, the encoding of bacterial virulence factors (e.g., bacterial toxins) occurs with temperate phages [88], thereby increasing bacterial virulence and creating completely new phenotypes, a process termed lysogenic conversion [84]. Despite their clear potential, phages have not been implemented globally in modern medicine, except for in Poland, Russia, and Georgia, where their use has been ongoing for decades [90].

### 3.2. Phages in Food Production and Animal Therapy Contributing to One Health

Phages represent a major problem for the dairy industry (and any industry dependent on bacterial growth and metabolic activities) in relation to fermentation procedures where they are present within the bacterial starter culture, causing cell death during the process. The industry relies on practical approaches to control phages, including modified factory design, increased sanitation, adequate ventilation, improved starter medium, and culture rotation [91]. Phages in animal therapy, however, have been primarily investigated in food-producing animals and in controlling foodborne pathogens associated with infectious disease. Foodborne diseases, which are largely bacterial, result in 420,000 deaths and an estimated 600 million cases of foodborne infections annually [58]. Foodborne disease represents a key area where One Health can successfully reduce the incidence of human disease and zoonosis. Indeed, phages are implemented in food processing in Canada, Israel, and the USA to target zoonotic pathogens, including *Listeria monocytogenes*, *Salmonella* species, and *E. coli* 0157:H7 [92]. ListShieldTM and SalmoFreshTM (Intralytix Inc.) commercial phages have been explored for the control of *Listeria monocytogenes* and *Salmonella enterica*, respectively, and are currently generally recognized as safe (GRAS) for use in the food industry [93]. Proteon Pharmaceuticals has developed a commercial phage product, BAFADORR, to eliminate *Pseudomonas* and *Aeromonas* infections in aquaculture, as intensive fish farming is prone to bacterial disease outbreaks. Phage therapy has been investigated to control *Salmonella* in chickens and pigs, as well as avian pathogenic *E. coli* (causative agent of colibacillosis) and *Campylobacter* in poultry, with varying degrees of success [94]. Intralytix has also marketed a phage product (INT-401TM) for treating *Clostridium perfringens*, the causative agent of necrotic enteritis in poultry [93]. Bacteriophage therapy demonstrated efficacy at preventing septicemia and meningitis, such as infection in calves caused by *E. coli* [95].

Studies investigating the efficacy of phage therapy against *A. baumannii* infection in mice demonstrated 2.3-fold increased survival in the treated vs. untreated group [96]. A novel lysin from *A. baumannii* prophages effective against clinical MDR isolates capable of curing fatal infectious disease in mice has also been characterized [91]. Inhaled phages treated *P. aeruginosa* hemorrhagic pneumonia in a mink model, *A. baumannii*- and *Klebsiella pneumoniae*-induced pneumonia in mice, and fatal *E. coli* respiratory infection in chickens [85]. The control of mastitis (intramammary infections IMIs) in cattle is an ongoing challenge, as incidence of mastitis causes animal morbidity, reduced milk yield, reduced milk quality, fertility issues, and substantial economic losses annually. While mastitis is caused by numerous pathogens (e.g., *E. coli, Enterococcus, Pseudomonas*, and *Streptococcus*), *Staphylococcus aureus*, however, is associated with chronic, contagious infections that are difficult to treat [64]. Furthermore, subclinical mastitis can lead to the bacterial contamination of dairy products and infectious disease in consumers. Mastitis is currently treated with a range of antibiotics and animal culling in extreme cases, and therefore is a bovine disease where One Health must be implemented to protect animal and human health. Phages may offer an alternative therapeutic option; research assessing the use of a 4-phage cocktail against 36 mastitis-associated *E. coli* isolates showed a 3- to 5-log decrease in *E. coli* in raw milk [97]. Studies have shown that the lysin produced by phage MR11 and intraperitoneal phage (ENB6) injections are active against MRSA [98] and VRE infections in mice [99]. Phage therapy against extracellular bacteria has been demonstrated by many studies; however, studies on intracellular bacteria are limited, which reside within host cells, such as in *Brucella, Chlamydia*, or Mycobacteria infections [95]. *Mycobacterium avium* subspecies paratuberculosis (MAP) is the causative agent of paratuberculosis or Johne’s disease (JD) in cattle, a chronic granulomatous wasting gastroenteritis that closely resembles Crohn’s disease (CD) in humans [97]. MAP is transmitted within herds vertically through contaminated milk and colostrum or horizontally through contaminated feed. The link between MAP and autoimmune bowel disease in humans is a topic of ongoing debate, with contaminated milk being a possible source of exposure to the pathogen. As such, MAP may represent a serious zoonotic pathogen causing chronic incurable disease in humans. Early detection and isolation of infected animals is essential for preventing disease transmission within the herd and zoonotic transmission via dairy food, which is again a key aspect of One Health. Mycobacteriophages (phages of mycobacterium) can be used in the detection of disease-causing mycobacteria, such as MAP in both JD in cattle [100] and CD in humans. Parental delivery is often used successfully for phage delivery in animal models, and intraperitoneally, intramuscularly, subcutaneously, and intravenously in animals and humans [85].

### 3.3. The Issue of Phages Promoting Pathogenesis and AMR

Temperate phages conducting a lysogenic cycle can insert phage genomes coding for toxins and other critical virulence factors into their bacterial host, increasing the bacterial pathogenesis to humans. This lysogenic conversion is necessary to ensure the survival of the bacteria and the virus in nature [84], as viruses are dependent on their host species. In this capacity, phages also modify the gene expression of host cells to prevent superinfection by other phages [101], thereby contributing to the phage’s specificity for bacterial species. Phages promote bacterial diversity as vectors for gene sharing via transduction, where genes are integrated into the bacterial chromosome. The integrated phage genome (prophage) is transmitted to the bacterial daughter cells as it replicates as a part of the host genome [102]. The new bacterial phenotypes possess immunity to phage superinfection, resistance to other phages, tolerance to various stresses, pathogenicity, and antibiotic resistance [11]. Inserted genes can include antibiotic resistance genes and genes coding for toxins that increase bacterial virulence. Shiga-toxin-producing *E. coli* (STEC), *Vibrio cholerae*, and *E. coli* 0157:H7 carrying the Shiga toxin are associated with hemolytic uremic syndrome [69]. Additional phage-encoded toxins include cholera, botulinum, diphtheria, and streptococcal pyrogenic exotoxins [63]. Phages gamma and C1 encode key virulence factors of *Corynebacterium diphtheriae* and *Clostridium botulinum* [103]. The transmission of ARGs by phages has been observed in numerous zoonotic bacteria, such as *Salmonella*, *Clostridium*, *Streptococcus*, *Staphylococcus*, and *Bacillus.* Approximately 75% of *Streptococcus pyogenes* isolates contain one or more prophages, many of which have the potential to perform transduction [81]. This transduction of ARGs includes an efflux pump that confers resistance to macrolide antibiotics. Phage P1 has demonstrated ability to transfer the β-lactamase gene between *E. coli* strains, resulting in multiple antimicrobial resistances [93]. Additionally, resistance genes such as *blaTEM* (βlactams), *qnrS* (fluoroquinolones), *ermB* (macrolides), *sulI* (sulphonamides), and *tetW* (tetracyclines) were identified in the virome of activated sludge and environmental water samples [104]. Phages used in therapy should not contain any genes that encode virulence factors or toxins. There is an increasing number of phage-encoded virulence factors being identified involving a wide range of genes, including ADP–ribosyl transferase toxins, superantigens, LPS modifying enzymes, type III effector proteins, detoxifying enzymes, hydrolytic enzymes, and proteins conferring serum resistance [103]. Stress such as starvation, environmental changes, and the presence of antibiotics can induce the expression of these prophage genes in bacterial species; consequently, antibiotic therapy can ultimately aggravate the infectious disease state [81].

### 3.4. Antifungal Phages, Mycosis, and One Health

Fungal dermal and invasive infections are increasing globally, with many species demonstrating antifungal resistance. Indeed, current antifungal therapeutics have other issues, such as severe toxicity to mammalian cells relating to hepatotoxicity and nephrotoxicity. Alternative, effective, safe antifungal agents are also needed to combat the increasing number of fungal species identified as causative agents of disease. Moreover, the zoonotic nature of respiratory and dermal fungal pathogens is common knowledge, where neglected fungal disease represents a serious threat to human and animal health. Immunocompromised patients are particularly at risk of increased morbidity and mortality from fungal infectious disease. As such, zoonotic fungal infections must also be considered under One Health in food-producing and companion animals. Indeed, emerging fungal infections are often neglected diseases, such as Paracoccidioidomycosis, Adiaspiromycosis, and lobomycosis, where zoonosis is often undetermined. Implementing the One Health approach is vital for preventing the emergence and re-emergence of such fungal diseases.

Some studies suggest that certain phages produced by *P. aeruginosa* may negatively impact fungal growth in *Aspergillus fumigatus* and *C. albicans* [105], zoonotic fungal species commonly associated with lung infection in cystic fibrosis patients. *C. albicans* is one of the most prominent nosocomial fungal infections globally and is associated with high mortality rates. Mycoviruses (viruses that selectively infect fungi) are phages of fungal species and have been identified for the major taxonomic classes of the fungi. The majority of these characterized mycoviruses have dsRNA genomes and are grouped into five families: *Partitiviridae, Totiviridae, Chrysoviridae, Reoviridae*, and *Hypoviridae* [106]. As with bacteriophages, mycoviruses can induce toxin production within the host fungi. For example, the filamentous basidiomycete fungus, *Ustilago maydis* [107], secretes a toxin encoded by cytoplasmic dsRNA mycoviruses, which can kill non-virally infected strains of the same fungus [108]. Toxin-producing yeasts have immunity to their own toxins, while being susceptible to those produced by other fungal species. Such dsRNA mycoviruses have been detected in *Saccharomyces cerevisiae, Hanseniaspora uvarum*, and *Zygosaccharomyces bailii*. Furthermore, the toxin isolated from *Z. bailii*, referred to as zygocin, kills *C. albicans, C. glabrata*, and *C. tropicalis* yeast cells [107] and appears to be more effective than the current antifungal agents clotrimazole or miconazole [109]. This antifungal protein toxin excreted by virally infected *Z. bailii* shows promise as an antifungal agent due to its broad-spectrum activity against a range of pathogenic and non-pathogenic zoonotic yeasts and filamentous fungi [110].

## 4. Conclusions

As microbial species become increasingly drug resistant, there is a dire need to find alternative therapy options for mitigating and controlling infectious diseases to protect human and animal health. Antibiotic-resistant zoonotic pathogens in the food chain and the re-emergence of zoonotic pathogens are global public health issues. The current antibiotic crisis necessitates the development of alternative strategies to fight drug-resistant bacterial species. Bacteriophages have been implemented in veterinary applications for decades, but their use in human medicine is limited and still requires clinical research. Studies have shown phage application in successfully treating zoonotic pathogens, including *Escherichia coli, Salmonella, Campylobacter, Pseudomonas aeruginosa, Klebsiella pneumoniae, Staphylococcus aureus, Streptococcus pyogenes*, and *Listeria monocytogenes*. Bacteriophages may also offer a means of controlling emerging and re-emerging pathogens, where 75% of emerging infectious disease are zoonotic according to the CDC. Key advantages include bacterial strain specificity and potency, with limited immune reactions induced in the patient. Indeed, phages are currently used as delivery vehicles for protein pharmaceutical ingredients and DNA vaccines. The phages’ self-propagation in vivo and their self-limiting nature following the clearance of the bacterial host also makes them attractive for combating infectious disease. As antimicrobial agents, however, their pharmacokinetics and pharmacodynamics need to be fully elucidated to maximize treatment options. Additionally, the evolution of bacterial resistance to phages must be understood if they are to be implemented therapeutically. While issues such as formulation, stability, and specificity must be overcome, initial studies indicate that phages have great potential for antimicrobial therapy in both human and animal medicines, thereby contributing to One Health. Antimicrobial phage therapy may be the way forward in combatting infectious disease and multidrug resistance.

## Figures and Tables

**Table 1:** Medically important bacterial species, their resistance mechanisms, therapeutics where resistance is evident, and associated morbidity.

Bacterial Species	Resistance Mechanisms	Therapeutics	Morbidity
**Gram-negative**			
Klebsiella pneumoniae *	Mutations in chromosomal genes, horizontal gene transfer (HGT) [20], efflux pump, ESBL production, intrinsic resistance [21] Reduced access to bacterial targets [22]	Carbapenem, third-generation cephalosporin [2]	Gastroenteritis, hemorrhagic diarrhea, Lipopolysaccharide-induced septic shock [23], deep wound infections, osteomyelitis, respiratory infections, bacteremia [22], enteric pathogenicity [21,24], nosocomial transmission
Acinetobacter baumannii *		Multi drug resistant—penicillins, cephalosporins, fluroquinolones, and aminoglycosides [25], multi drug resistant above plus carbapenem	
Escherichia coli *		Carbapenem, third-generation cephalosporin [2]	
Pseudomonas aeruginosa *	Broad intrinsic antimicrobial resistance, efflux pump, extended spectrum beta lactamase production, HGT, psychrotrophic [21]	Multidrug resistant, carbapenem, aminoglycosides, cephalosporins	Fatalities, nosocomial infections—urinary tract infections (UTIs), bacteremia, chronic airway infection in cystic fibrous patients [26]
Neisseria gonorrhea **	Cumulative chromosomal mutations in different genes related to cell wall biosynthesis [27], TetM protein conferring tetracycline resistance [28]	Azithromycin, third-generation cephalosporins, fluoroquinolones, sulfonamides, penicillin, tetracycline [27]	Gonorrhea, sexually transmitted disease (STI) and drug resistance
Salmonella species **	Gene mutation—DNA gyrase, efflux pump [29], alterations to outer membrane proteins [30], extended-spectrum cephalosporinases	Fluoroquinolone ciprofloxacin [29], ampicillin, chloramphenicol, sulfamethoxazole–trimethoprim tetracycline and streptomycin [31]	Foodborne disease, gastroenteritis, enteric fever, typhoid [32]
Helicobacter pylori **	Cytotoxin-associated gene A (*cagA*) [33] mutations, DNA gyrase [34]	Clarithromycin, metronidazole, levofloxacin [33], amoxicillin, and tetracycline [35]	Peptic ulcer disease, lymphoma, gastric adenocarcinoma [35]
**Gram-positive**			
VRE **	β-lactamase, RNA methyltransferase, mutations in genes altering membrane structure [36]	Vancomycin, ampicillin, cephalosporins, aminoglycosides, daptomycin [36], low levels of intrinsic resistance to the quinolones	Nosocomial UTIs, immunosuppressed persons, bacteremia, bacteriuria [37], endocarditis, peritonitis [37]
MRSA **	Heat-stable staphylococcal enterotoxin production [21], altered penicillin-binding proteins (PBPs) [38]	Methicillin, amoxicillin, penicillin, oxacillin, cephalosporins, intrinsically resistant to the carbapenems [39]	Toxic shock syndrome, pneumonia, mastitis impetigo, cellulitis, osteomyelitis, endocarditis, bacteremia [38]
*Clostridioides difficile*	Erythromycin ribosomal methylase (*erm*) gene [40]	Aminoglycosides, lincomycin, tetracyclines, erythromycin [40], clindamycin, penicillin, cephalosporins, fluoroquinolones [41]	Nosocomial mortalities, pseudomembranous colitis, toxin-mediated disease [41]
Streptococcus pneumoniae ***	The erm(B) gene, altered PBPS [42], mutations of DNA gyrase gene, *tet(M)* and *tet(O)* genes [43]	Beta-lactam antibiotics [42], macrolides, lincosamides, fluoroquinolones, tetracyclines, trimethoprim–sulfamethoxazole [43]	Community-acquired pneumonia, meningitis, sepsis, bacteremia, and otitis media [43]
**Acid Fast**			
*Mycobacterium tuberculosis*	Mutations in the *embB* gene [44], mutations in the *pncA* gene [45]	Ethambutol, rifampicin, isoniazid, pyrazinamide [45]	Tuberculosis (TB), multi drug resistant TB [44]
*Mycobacterium avium complex* *(M. avium, M. intracellulare)*	Lipid-rich cell wall [46], gene mutations in PBPs, *embB*, *embR*, *rpsL* [47], efflux pump, β-lactamases	Intrinsic multidrug resistance [46], macrolides [48], clarithromycin [48]	Mycobacterium avium-intracellular infection, lung disease [49], disseminated infection (usually associated with AIDS), lymphadenitis, localized cutaneous infection with tenosynovitis [47]

* Critically important pathogens on the WHO priority pathogen list. ** Highly important pathogens on the WHO priority pathogen list. *** Medium importance pathogens on the WHO priority pathogen list.

**Table 2:** Virus order, family, viral examples, features, target species, and related taxonomy.

Order	Family	Bacteriophage	Features	Target Species
Caudovirales	*Myoviridae*	T2, T4, vBSM-A1 [64]	dsDNA, linear [58], NE, contractile tail [60]	*Escherichia coli, S. aureus*
	*Siphoviridae*	λ, T1, T5, ΦAPCM01	dsDNA, linear, NE, non-contractile tail [62]	*S. mutans* [65]
	*Podoviridae*	T7, vBSP-A2, PFpW-6		*E. coli, S. aureus* [64]
Ligamenvirales	*Lipothrixviridae*	TTV1, SIFV	Helical, dsDNA, linear, E, rod-shaped	Thermophilic Archaea
	*Rudiviridae*	SIRV1, AVF-1	Helical, dsDNA, linear, NE, bottle shaped	Hyperthermophilic Archaea
Non grouped	*Inoviridae*	Fd, pf1, Vf33 [66]	Filamentous, ssDNA, NE, circular	Enterics, *Pseudomonas, Vibrio* [67]
	*Microviridae*	PhiX174	Polyhedral ssDNA [66]	Enterobacteria
	*Tectiviridae*	PRD1	Linear dsDNA	Gram-negative bacteria
	*Corticoviridae*	PM2	Highly supercoiled, dsDNA, NE, circular [66]	
	*Cystoviridae*	Phi6	dsRNA, linear, lipoprotein envelope, spherical	*Pseudomonas* species-specific
	*Leviviridae*	MS2 [66]	Polyhedral ssRNA, linear	Enterics, *Acinetobacter, Pseudomonas*
	*Ampullaviridae*	Acidianus bottle-shaped virus	dsDNA, linear, E, bottle-shaped	Archaea
	*Bicaudaviridae*	Acidianus two-tailed virus	dsDNA, circular, NE, lemon-shaped	Hyperthermophilic archaea
	*Clavaviridae*	Aeropyrum pernix bacilliform virus 1.	NE, rod shaped, dsDNA, circular	Aeropyrum pernix
	*Fuselloviridae*	Sulfolobus spindle-shaped virus 1, SSSV2, SSSV3	Superhelical dsDNA [67], circular	Thermophilic Archaea
	*Plasmaviridae*	Acholeplasma virus L2	Superhelical dsDNA, E, pleomorphic	Acholeplasma laidlawii
	*Globuloviridae*	Pyrobaculum spherical virus	Helical, E, dsDNA, linear [62]	Hyperthermophilic archaea, genera Pyrobaculum, and Thermoproteus

NE = non-enveloped; E = enveloped.
